# Long non-coding RNA-small nucleolar RNA host gene 7 regulates inflammatory responses following spinal cord injury by regulating the microRNA-449a/TNF-α-induced protein 3-interacting protein 2 axis

**DOI:** 10.1080/21655979.2022.2061294

**Published:** 2022-04-20

**Authors:** Chunlei He, Jianhua Xiao, Yongjun Ye, Shiqiao Huang, Yanchun Zhong, Lulin Liu, Wuyang Liu, Sheng Liu

**Affiliations:** Department of Orthopedics, The First Affiliated Hospital of Gannan Medical University, Ganzhou, Jiangxi, P.R. China

**Keywords:** Spinal cord injury, microRNA, long non-coding RNA, TNF-α-induced protein 3-interacting protein 2, NF-κB pathway

## Abstract

The current study aimed to explore the anti-inflammatory effects of long non-coding RNA-small nucleolar RNA host gene 7 (lncRNA-SNHG7) and its mechanism in spinal cord injury (SCI) models. SCI models were established both *in vivo* and *in vitro*. Reverse transcription-quantitative PCR was performed to determine the expression levels of lncRNA-SNHG7 in SCI models. Bioinformatics analysis and dual-luciferase reporter assays were carried out to confirm the interaction between lncRNA-SNHG7 with microRNA (miR)-499a and TNF-α-induced protein 3-interacting protein 2 (TNIP2). In addition, cell viability, apoptosis, and the secretion of inflammatory cytokines were assessed by 3-(4,5-dimethyl-2-thiazolyl)-2,5-diphenyl-2-H-tetrazolium bromide (MTT) assay, flow cytometric analysis, and enzyme linked immunosorbent assay (ELISA), respectively. The results showed that lncRNA-SNHG7 was markedly downregulated in the SCI model group. LncRNA-SNHG7 directly bound to miR-499a, which in turn directly targeted TNIP2. In addition, TNIP2 was significantly decreased in SCI rats and lipopolysaccharide (LPS)-treated PC-12 cells. The *in vitro* results in PC-12 cells revealed that lncRNA-SNHG7 overexpression attenuated neuronal cell death and SCI-mediated inflammatory responses by regulating miR-449a expression. Furthermore, miR-499a knockdown inhibited LPS-induced PC-12 cell injury by targeting TNIP2. In conclusion, lncRNA-SNHG7 modulates the apoptosis and inflammation of PC-12 cells by regulating the miR-449a/TNIP2/NF-κB signaling pathway.

## Graphical Abstract

**Figure:**
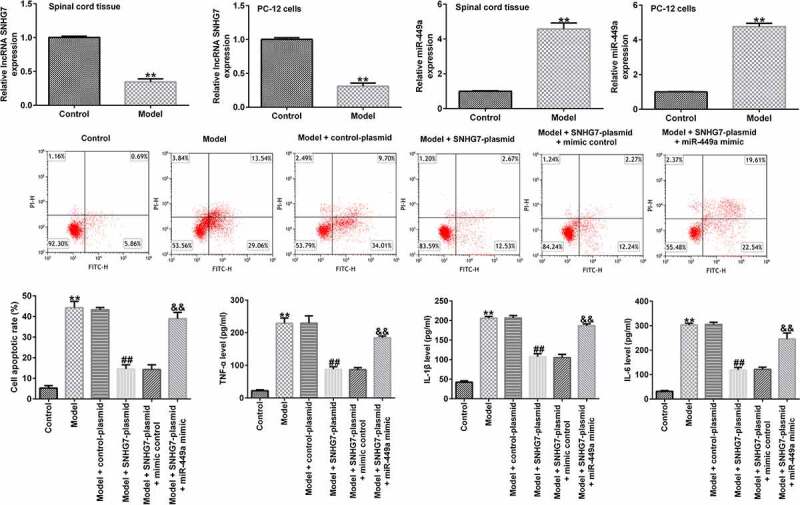

## Introduction

Spinal cord injury (SCI) is a serious neurological condition that may result in severe disorders of both sensory and motor neurons [1–4]. SCI is associated with the development of severe physical and psychological disorders in affected patients and poses a major socioeconomic burden [5]. The prevention, therapy, and recovery from SCI have become important topics in the medical field. Although some progress has been made with respect to the pathogenesis of SCI, the exact mechanism remains unclear. Therefore, further research is urgently needed [6,7]. SCI may lead to complete and permanent loss of neurological function. Following injury, a series of pathophysiological events may occur, including loss of blood supply, inflammation, and demyelination, thus promoting the development of an adverse microenvironment in the injured area, which affects nerve regeneration and recovery [8,9]. Therefore, regulating inflammation in the early stages of SCI is considered a key approach for treating SCI [9,10].

Long non-coding RNAs (lncRNAs) are a novel class of RNA transcripts more than 200 nucleotides in length and narrow protein-coding functions [11]. LncRNA-SNHG7, a newly identified oncogene [12], has been shown to participate in heart remodeling, liver fibrosis, osteoarthritis, and ischemic stroke [13–16]. However, its role in SCI remains largely unknown.

MicroRNAs (miRNAs/miRs) are endogenous non-coding RNAs with length ranging from 20–25 nucleotides, which regulate specific gene expression at the post-transcriptional level [17]. The expression profiles of several miRNAs are altered following SCI [18–21]. Studies have shown that miRNAs are related to the regulation of the expression of numerous genes in SCI. The expression of these genes is closely associated with SCI-mediated inflammatory reactions, neuronal necrosis, and other pathological processes [22–26]. Previous studies have suggested that miR-499a is overexpressed in multiple types of central nervous system injuries, including traumatic brain injury and pulmonary fibrosis [27,28]. However, the effects of miR-499a on SCI have not been fully investigated.

NF-κB signaling is related to the inflammatory process by regulating the transcription of pro-inflammatory genes [29]. It has been reported that following stimulation of cells with TNF-α and lipopolysaccharide (LPS), IκBα is degraded and NF-κB is released into the nucleus. Overactive NF-κB signaling has been shown to be associated with numerous inflammatory and autoimmune diseases [30]. TNF-α-induced protein 3-interacting protein 2 (TNIP2), also known as ABIN2, is a binding partner of A20 and a negative regulator of NF-κB signaling. TNIP2 was shown to increase IKKα-mediated NF-κB activation and induce the transcription of NF-κB-related target genes by enhancing IKKα autophosphorylation and kinase activity [31]. Another study demonstrated that upregulation of TNIP2 suppressed the activation of the NF-κB signaling pathway, thus regulating the proliferation of PANC-1 cells [32].

In the present study, we hypothesized that lncRNA-SNHG7 affects SCI development via the regulation of miR-449a expression. Therefore, the present study was designed to examine the roles of lncRNA-SNHG7/miR-449a in an SCI rat model and an SCI cellular model, and to determine whether the lncRNA-SNHG7/miR-449a axis is associated with the occurrence and progression of SCI via regulation of inflammatory responses and neuronal apoptosis.

## Materials and methods

### Animals

Healthy adult male Sprague-Dawley (SD) rats (6–10 weeks old; 230–300 g; n = 40) were purchased from the Animal Center of the General Hospital of Jinan Military Area Command (Jinan, China) and were placed in a standard environment (24 ± 1°C, 50–60% humidity, 12 h light/dark cycle). The rats were fed a standard laboratory diet and had free access to drinking water. This research protocol was approved by the Scientific Review Committee of The First Affiliated Hospital of Gannan Medical University.

### Establishment of the SCI rat model

SD rats were anesthetized with 4% pentobarbital (30 mg/kg) via intraperitoneal injection. Following anesthesia, a longitudinal incision was made on the midline of the back to expose the paravertebral muscles, followed by opening and rinsing with normal saline. A laminectomy was performed on the tenth vertebra to expose the dura mater. The SCI model was established by striking the T10 segment of the thoracic vertebrae with a 5 G rod from a height of 5.0 cm [33].

### Cell culture and LPS stimulation

PC-12 cells (cat. no. CRL-1573; ATCC) were grown in Dulbecco’s modified Eagle’s medium (DMEM; Gibco, Grand Island, NY, USA) containing 10% fetal bovine serum (FBS; Gibco, Grand Island, NY, USA) in a humidified incubator with 5% CO_2_ at 37°C. LPS induced PC-12 cell model has been widely used for *in vitro* investigation of SCI [34–36]. To generate the SCI *in vitro* model, PC-12 cells (10^6^ cells/mL) were treated with 100 ng/ml LPS for 4 h at 37°C, as previously described [34].

### miRNA transfection

Cells were cultivated in a 6-well cell culture plate (10^6^ cells/well) and were then transfected with 1ng lncRNA-SNHG7 plasmid (GenePharma Co., Ltd., Shanghai, China), 1ng control plasmid, 100 nM miR-449a mimics (Guangzhou Ribobio Co., Ltd, Guangzhou, China), 100 nM mimics control (Guangzhou Ribobio Co., Ltd), 50 nM miR-449a inhibitor (Guangzhou Ribobio Co., Ltd), 50 nM control inhibitor (Guangzhou Ribobio Co., Ltd), 0.2 µM control-small interfering RNA (control-siRNA; Santa Cruz Biotechnology, Dallas, TX, USA), or 0.2 µM si-TNIP2 (Santa Cruz Biotechnology, Dallas, TX, USA) using Lipofectamine® 2000 (Life Technologies Corporation, Savant, MA, USA), according to the manufacturer’s instructions.

### Dual-luciferase reporter analysis

TargetScan software was used to identify the potential target genes of miR-449a. The analysis predicted that TNIP2 could be directly targeted by miR-449a. The interaction between miR-499a and TNIP2 was verified using a dual-luciferase reporter assay [37]. The TNIP2 3’-untranslated region encompassing the wild-type (WT) or mutant (MUT) miR-449a-binding site was synthesized by genomic PCR and cloned into pMIR vectors (Ambion; Thermo Fisher Scientific Inc., Savant, MA, USA) to construct the TNIP2-WT or TNIP2-MUT reporter plasmids. Subsequently, TNIP2-WT or TNIP2-MUT and miR-449a mimics or mimics control were transfected into 293 T cells using Lipofectamine 2000® (Life Technologies Corporation, Savant, MA, USA), according to the manufacturer’s instructions. Following transfection for 48 h, the luciferase reporter assay system (Promega, Madison, MI, USA) was used to assess the luciferase activity according to the manufacturer’s instructions. The data are displayed as firefly luciferase activity normalized to *Renilla* luciferase activity.

### Western blot analysis

Protein expressions were determined using western blot assay in this study [38]. PC-12 cells (2 × 10^6^ cells/mL) were lyzed using RIPA lysis buffer (Beyotime, Shanghai, China) on ice, and the protein concentration was measured using a BCA assay (Beyotime, Shanghai, China). Protein samples (50 µg/lane) were mixed, boiled, centrifuged, and resolved by 10% sodium dodecyl sulfate polyacrylamide gel electrophoresis (SDS-PAGE) and transferred to polyvinylidene difluoride (PVDF) membrane (Millipore Sigma, St. Louis, MO, USA). After incubation with 5% nonfat milk in phosphate buffered saline (PBS)-Tween-20 for 1 h, the membrane was incubated with the following primary antibodies: anti-TNIP2 (1:1000; cat. no. ab205925), anti-phosphorylated (p)-p65 (1:1000; cat. no. ab76302), and anti-p65 (1:1000; cat. no. ab16502; all from Abcam, Cambridge, MA, UK). The membrane was then incubated with a secondary antibody (1:2000; cat. no. ab7090; Abcam) for 1 h at room temperature. The blots were assessed using ECL detection reagent (Beyotime, Shanghai, China) and analyzed using Image Lab software (version 4.0; Bio-Rad Laboratories Inc., Hercules, CA, USA).

### RT-qPCR analysis

Total cellular RNA was isolated from PC-12 cells and spinal cord tissues using TRIzol® reagent (Invitrogen; Thermo Fisher Scientific, Inc.) and reverse transcribed into cDNA with the PrimeScript™ RT Reagent Kit (TaKaRa, Beijing, China) following the manufacturer’s instructions. An ABI 7000 Real-Time PCR system (Applied Biosystems, USA) with SYBR® Green PCR Master Mix Kit (Takara, Beijing, China) was used to examine gene expression levels. Gene expression was quantified using the 2^−ΔΔCq^ method [39]. Primer sequences for PCR were listed as following:

miR-449a forward, 5′-CGCGCGTGGCAGTGTATTGTTA-3′;

reverse, 5′-ATCCAGTGCAGGGTCCGAGG-3′;

lncRNA-SNHG7 forward, 5′-GTGACTTCGCCTGTGATGGA-3′;

reverse, 5′-GGCCTCTATCTGTACCTTTATTCC-3′;

p65 forward, 5′-ATGTGGAGATCATTGAGCAGC-3′;

reverse, 5′-CCTGGTCCTGTGTAGCCATT-3′;

TNIP2, forward 5′-CTAAAGAGGCGGCAGGTCCCTC-3′;

reverse, 5′-CAAGATGACCTTCCAGTGAC-3′;

GAPDH forward, 5′-CATCATCCCTGCCTCTACTGG-3′;

reverse, 5′-GTGGGTGTCGCTGTTGAAGTC-3′;

U6 S, 5′-GGAACGATACAGAGAAGATTAGC-3′;

Stem-loop-R, 5′-CTCAACTGGTGTCGTGGAGTC-3′.

### MTT assay

For the MTT assay [40], each well was supplemented with 50 μL MTT reagent (cat. no. ab211091; Abcam, Cambridge, MA, UK). Following incubation for 3 h at 37°C, the absorbance at 570 nm was determined using an Infinite M1000PRO Tecan spectrophotometer (Tecan Group Ltd.).

### Flow cytometric (FCM) analysis

Following treatment, PC-12 cells were analyzed using a double staining apoptosis detection kit (Beyotime) [41]. Annexin V-fluorescein isothiocyanate (FITC) and PI were added to the PC-12 cell suspension and the cells were incubated for 30 min at 37°C in the dark according to the manufacturer’s instructions. Finally, the apoptotic cells were detected using a flow cytometer (BD Biosciences, San Diego, CA, USA).

### Detection of inflammatory factors

The secretion of IL-1β (cat. no. ab100704), and TNF-α (cat. no. ab100747), and IL-6 (cat. no. ab100712; all from Abcam, Cambridge, MA, UK) were measured using the corresponding ELISA kits according to the manufacturer’s instructions [42].

### Statistical analysis

Statistical analyses were conducted using SPSS 20.0. All results are expressed as mean ± standard deviation from three independent experiments. Mean differences between groups were evaluated using analysis of variance and Student’s *t*-test. Statistical significance was set at P < 0.05.

## Results

### lncRNA-SNHG7 was downregulated, and miR-499a was significantly upregulated in the in vivo and in vitro models of SCI

**Figure 1.**
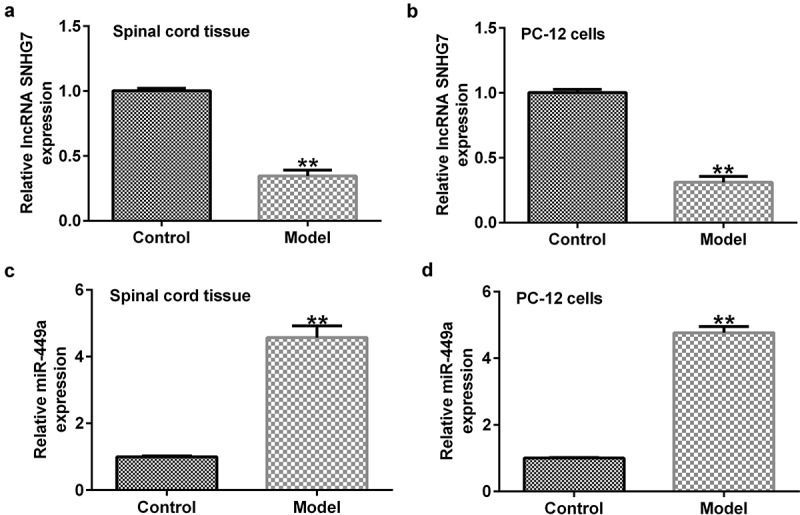
lncRNA-SNHG7 and miR-499a expression in rat and *in vitro* SCI models. Reverse transcription-quantitative PCR assay was carried out to determine the expression levels of (a and b) SNHG7 and (c and d) miR-449a in SCI rats and SCI *in vitro* cells models. **P < 0.01 vs. the NC group. lncRNA-SNHG7, long non-coding RNA-small nucleolar RNA host gene 7; miR-499a, microRNA-499a; SCI, spinal cord injury.

First, RT-qPCR was performed to evaluate the role of SNHG7 in SCI. As shown in Figure 1(a, b), SNHG7 was significantly downregulated in the SCI rat model and *in vitro* model compared to the control. Previous studies revealed that miR-449a directly targets SNHG7, and SNHG7 negatively regulates miR-449a expression. Therefore, it was hypothesized that SNHG7 may play an important role in SCI by regulating miR-449a. We then determined the levels of miR-449a in the SCI rat and cell models and found that the levels of miR-499a in the *in vivo* and *in vitro* SCI models were higher than those in the control group (Figure 1(c, d)).

### lncRNA-SNHG7 negatively regulates miR-499a expression in PC-12 cells

**Figure 2.**
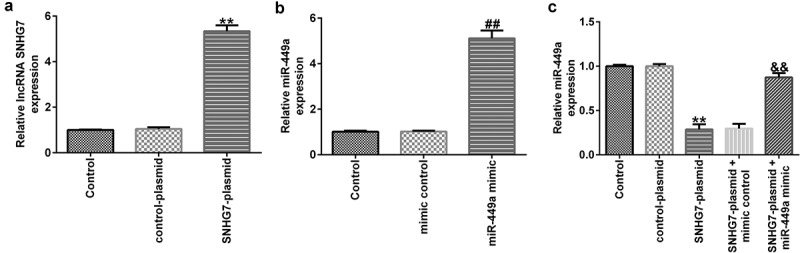
lncRNA-SNHG7 negatively regulates miR-499a expression in PC-12 cells. SNHG7 plasmid, control plasmid, miR-449a mimics, mimics control, lncRNA-SNHG7 plasmid + mimics control or lncRNA-SNHG7 plasmid + miR-449a mimics were transfected into PC-12 cells for 48 h. (a-c) lncRNA-SNHG7 and miR-449a levels in different groups were determined by qRT-PCR. * P < 0.05, **P < 0.01 vs. control.

Next, to examine whether lncRNA-SNHG7 could negatively regulate miR-499a levels in PC-12 cells, lncRNA-SNHG7 plasmid, control plasmid, miR-449a mimics, mimic control, lncRNA-SNHG7 plasmid + mimics control, or lncRNA-SNHG7 plasmid + miR-449a mimics were transfected into PC-12 cells for 48 h, and gene expression was determined using RT-qPCR. We found that transfection of PC-12 cells with lncRNA-SNHG7 plasmid significantly upregulated lncRNA-SNHG7 expression, compared to cells transfected with the control plasmid (Figure 2(a)). In addition, compared to the control group, the levels of miR-449a were notably increased in PC-12 cells following miR-449a mimic transfection (Figure 2(b)). As expected, lncRNA-SNHG7 overexpression markedly downregulated miR-449a in PC-12 cells, whereas this effect was reversed following miR-449a overexpression (Figure 2(c)).

### miR-499a abolished the influence of lncRNA-SNHG7 in the in vitro SCI model

**Figure 3.**
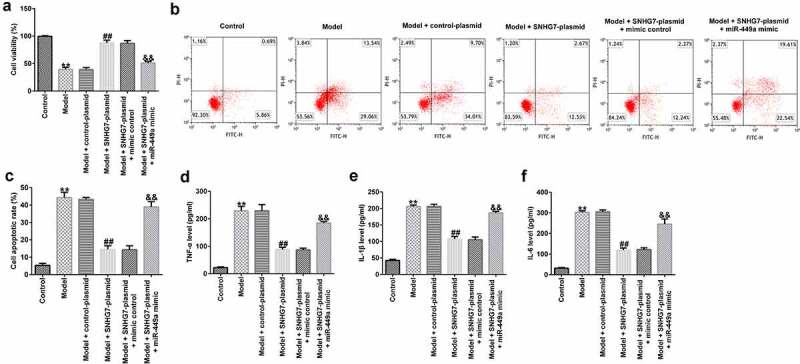
miR-499a abolishes the influences of lncRNA-SNHG7 in a cell model of SCI. PC-12 cells were transfected with lncRNA-SNHG7 plasmid, control plasmid, lncRNA-SNHG7 plasmid + mimics control or lncRNA-SNHG7 plasmid + miR-449a mimics for 48 h and were then treated with 100 ng/ml lipopolysaccharide for 4 h. Cell viability, apoptosis, and the secretion of inflammatory cytokine were determined using MTT (a), flow cytometry analysis (b and c), and ELISA assay (d-f), respectively. * P < 0.05, **P < 0.01 vs. control.

To further assess the functions of lncRNA-SNHG7 in SCI *in vitro*, PC-12 cells were transfected with lncRNA-SNHG7 plasmid, control plasmid, lncRNA-SNHG7 plasmid + mimics control, or lncRNA-SNHG7 plasmid + miR-449a mimics for 48 h and then treated with 100 ng/ml LPS for 4 h at 37°C. Subsequently, MTT assay, FCM, and ELISA were performed to determine cell viability, cell apoptosis, and the secretion of IL-1β, TNF-α, and IL-6, respectively. As shown in Figure 3, cell viability was significantly attenuated (Figure 3(a)), cell apoptosis was elevated (Figure 3(b, c)), and the secretion of inflammatory factors (Figure 3(d–f)) was notably enhanced in the LPS treatment group compared to that in the control group. Furthermore, in comparison with the model + control plasmid group, lncRNA-SNHG7 overexpression significantly enhanced cell viability, decreased cell apoptosis, and reduced inflammatory factor release. However, these effects were markedly abrogated following miR-449a overexpression, suggesting that miR-499a may abolish the functions of lncRNA-SNHG7 in LPS-treated PC-12 cells.

### TNIP2 directly interacts with miR-449a

**Figure 4.**
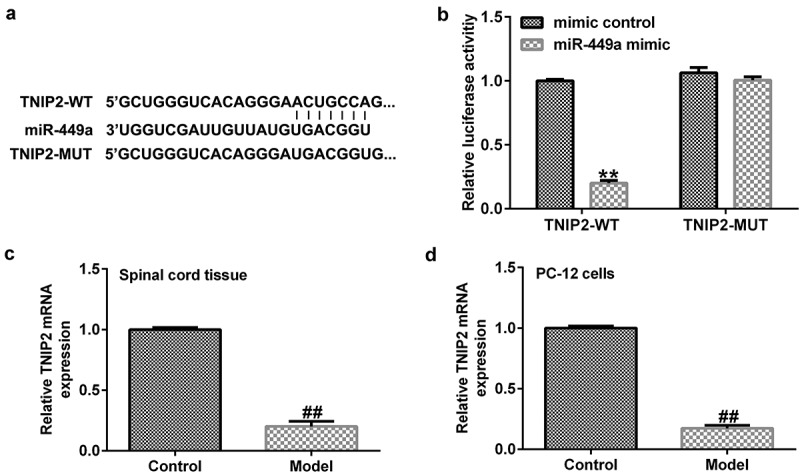
TNIP2 expression in rat and *in vitro* SCI models. (a) A schematic of TNIP2 binding site in miR-499a 3’-UTR. (b) The interaction between miR-449a and TNIP2 were verified using dual-luciferase reporter assay. (c and d) qRT-PCR analysis of TNIP2 in rat and *in vitro* SCI models. * P < 0.05, **P < 0.01 vs. control.

To investigate the regulatory mechanism of miR-499a in SCI, the TargetScan bioinformatics prediction tool was used to predict the latent targets of miR-499a. Figure 4(a) illustrates the relationship between miR-499a and TNIP2. A dual-luciferase reporter assay confirmed the specific regulatory association between miR-449a and TNIP2. We found that miR-449a overexpression attenuated TNIP2-WT luciferase activity compared to the mimic control group (Figure 4(b)), revealing that TNIP2 directly interacts with miR-449a. Following confirmation of the interaction between TNIP2 and miR-499a, we sought to examine whether the expression of TNIP2 was altered during SCI. Western blot analysis and RT-qPCR indicated that TNIP2 was markedly downregulated in SCI, compared to the control (Figure 4(c, d)). In summary, our findings suggest that miR-499a is involved in the development of SCI by regulating TNIP2 expression.

### miR-449a negatively regulates TNIP2 expression in PC-12 cells

**Figure 5.**
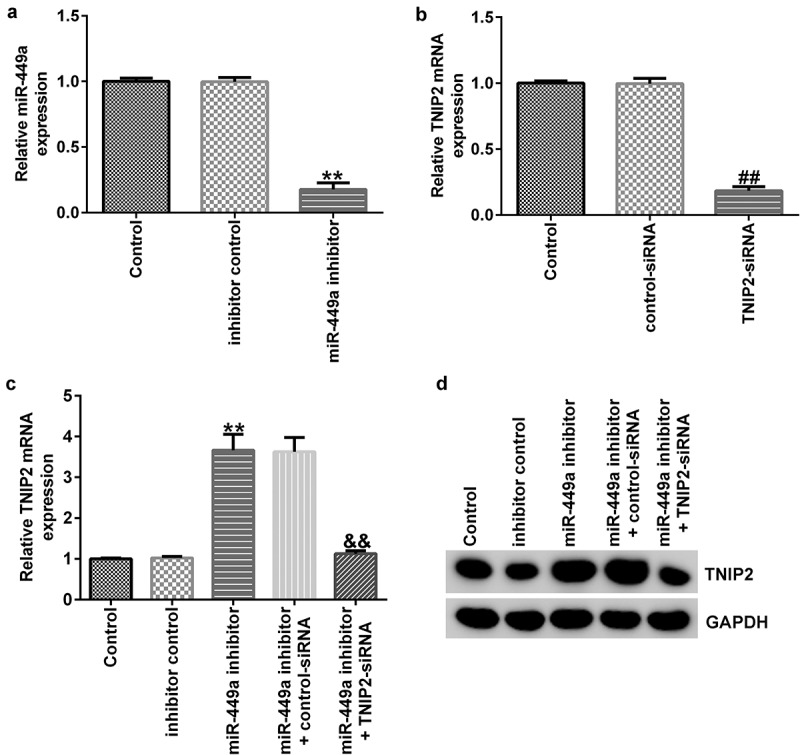
miR-449a negatively regulates TNIP2 expression in PC-12 cells. miR-449a inhibitor, inhibitor control, control-siRNA or si-TNIP2 were transfected into PC-12 cells for 48 h. (a) miR-449a expression in different groups was determined using RT-qPCR. (b and c) TNIP2 mRNA expression in different groups was determined using RT-qPCR. (d) Western blot analysis of TNIP2 protein expression. * P < 0.05, **P < 0.01 vs. control.

To explore the effect of miR-499a on TNIP2 expression in PC-12 cells, miR-449a inhibitor, inhibitor control, control-siRNA, si-TNIP2, miR-449a inhibitor + control-siRNA, or miR-449a inhibitor + si-TNIP2 were transfected into PC-12 cells for 48 h. Levels of miR-449a and TNIP2 were then assessed by RT-qPCR. As shown in Figure 5(a), miR-449a was downregulated in miR-449a inhibitor-transfected PC-12 cells. In addition, TNIP2 silencing notably decreased TNIP2 mRNA levels in PC-12 cells, compared to the control-siRNA transfected cells (Figure 5(b)). Furthermore, transfection with miR-449a inhibitor significantly enhanced TNIP2 mRNA expression and protein level in PC-12 cells, whereas this effect was reversed following TNIP2 knockdown (Figure 5(c, d)).

### The miR-449a inhibitor suppressed LPS-stimulated PC-12 cell damage by targeting TNIP2

**Figure 6.**
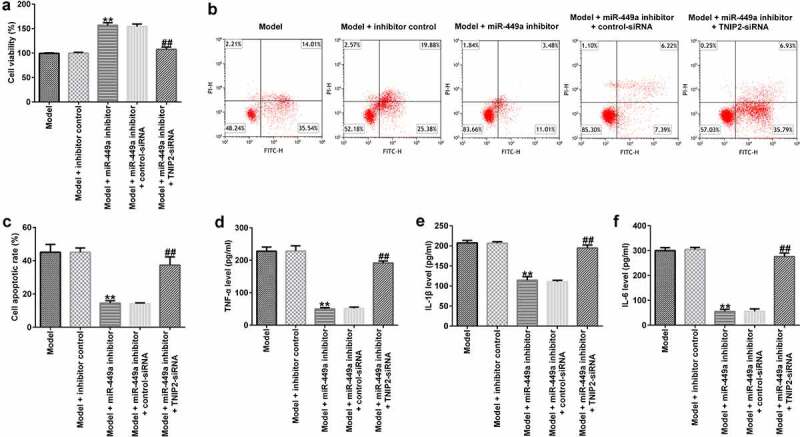
TNIP2 silencing abolishes the effects of miR-449a inhibitor in SCI. PC-12 cells were transfected with miR-449a inhibitor, inhibitor control, control-siRNA or si-TNIP2 for 48 h, and treated with 100 ng/ml lipopolysaccharide for an additional 4 h. Subsequently, cell viability, apoptosis, and secretion of inflammatory cytokines were determined using MTT assay (a), flow cytometric analysis (b and c), and ELISA assay (d-f), respectively. * P < 0.05, **P < 0.01 vs. control.

To identify the regulatory role of miR-449a in SCI, PC-12 cells were transfected with miR-449a inhibitor, inhibitor control, miR-449a inhibitor + control-siRNA or miR-449a inhibitor + si-TNIP2 for 48 h and then induced with 100 ng/ml LPS for an additional 4 h. Then, MTT and FCM assays were carried out to evaluate cell viability and apoptosis, respectively. The results revealed that the miR-449a inhibitor significantly enhanced cell growth (Figure 6(a)) and notably attenuated cell apoptosis (Figure 6(b, c)), compared to the model + inhibitor control group. Additionally, transfection with miR-449a inhibitor significantly reduced the secretion of inflammatory cytokines in LPS-induced PC-12 cells (Figure 6(d–f)). However, the aforementioned miR-449a inhibitor-mediated effects on LPS-induced PC-12 cells were reversed following TNIP2 knockdown.

### The NF-κB pathway is related to the regulatory mechanism of miR-449a in SCI in vitro

**Figure 7.**
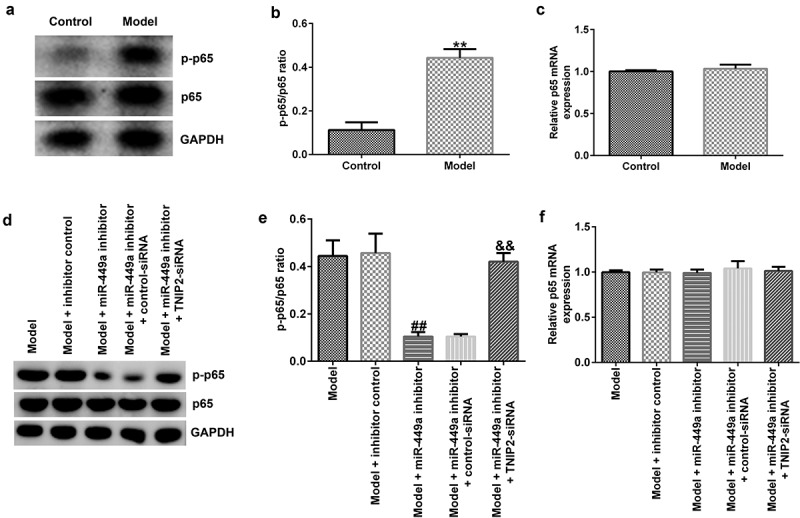
NF-κB pathway activation in SCI model *in vitro*. PC-12 cells were transfected with miR-449a inhibitor, inhibitor control, control-siRNA or si-TNIP2 for 48 h, and stimulated with 100 ng/ml lipopolysaccharide for 4 h. (a) Determination of p-p65 and p65 protein expression using western blot assay. (b) p-p65/p65 ratio. (c) RT-qPCR analysis of p65 mRNA levels. (d) Determination of p-p65 and p65 protein expression using western blot assay. (e) p-p65/p65 ratio. (f) p65 mRNA expression was measured using RT-qPCR. * P < 0.05, **P < 0.01 vs. control.

Previous investigations have revealed that the NF-κB pathway is activated during SCI. We finally explored whether NF-κB pathway is involved in the mechanism of lncRNA-SNHG7 in SCI. In the present study, compared to the control, the levels of p-p65 (Figure 7(a)) and p-p65/p65 ratios (Figure 7(b)) were significantly increased in the model group, indicating activation of the NF-κB pathway. However, the mRNA expression levels of p65 in the control and model groups were not significantly different (Figure 7(c)). Further analysis demonstrated that the increased levels of p-p65 (Figure 7(d)) and p-p65/p65 ratio (Figure 7(e)) were significantly reduced by the miR-449a inhibitor, whereas these changes were abolished by TNIP2 knockdown. No obvious changes were observed in the mRNA levels of p65 (Figure 7(f)).

## Discussion

SCI is widely encountered worldwide, accounting for > 2 million cases globally [43]. SCI poses a major burden on patients, their families, and society. Although the pathological mechanism of SCI has been studied extensively, the clinical application of neuroprotective and regenerative therapies remains limited [44]. In recent years, lncRNAs have become a new target as they regulate several physiological functions and mediate numerous neurological diseases [45]. Emerging studies have confirmed that lncRNAs are involved in the occurrence and development of SCI [45–47]. In our study, the levels of lncRNA-SNHG7 were determined in *in vivo* and *in vitro* models of SCI, and their function was evaluated *in vitro*.

LncRNA-SNHG7 was significantly downregulated in SCI rats and *in vitro* SCI models, thus supporting its potential role in the development of SCI. Previous studies demonstrated that lncRNA-SNHG7 directly binds to miR-449a to negatively regulate its expression [48,49]. The complex regulatory network of miRNAs can regulate the expression of multiple genes by a single miRNA, and the function of each miRNA varies among different cells and even among different cell states [18]. The effects of several miRNAs in SCI have been previously reported. For instance, Wang *et al*. [50] confirmed that miR-223-3p negatively regulates the receptor-interacting protein kinase 3 necroptotic signaling cascades and secretion of inflammatory factors to significantly alleviate SCI. Additionally, Ji *et al*. [51] suggested that LPS downregulates miR-132, which in turn attenuates LPS-stimulated inflammatory cell damage by regulating TRAF6 and inhibiting the activation of the NF-κB and MEK/ERK signaling pathways. Therefore, we hypothesized that lncRNA-SNHG7 plays a significant role in SCI by regulating miR-449a. The results verified that miR-449a was upregulated in SCI both *in vivo* and *in vitro*.

Pathological changes after SCI mainly include primary and secondary injuries [52]. Primary injury is irreversible, whereas secondary injury mainly causes aggravation of neurological dysfunction [53]. Secondary injury is reversible and can be adjusted, and is characterized by inflammation and cell apoptosis [54]. In this study, to determine the functions of lncRNA-SNHG7 in SCI, 100 ng/ml LPS was applied for 4 h to induce PC-12 cells prior to use as SCI cell models. Next, the effect of lncRNA-SNHG7 on LPS-stimulated PC-12 cell damage was investigated. Our results showed that miR-499a abolished the protective role of lncRNA-SNHG7 in LPS-induced PC-12 cells. To further explore the regulatory mechanism of miR-449a in SCI *in vitro*, the targets of miR-449a were identified. The results revealed that TNIP2 was a direct target and was negatively regulated by miR-449a. Furthermore, it was confirmed that cell transfection using si-TNIP2 reversed the functions of the miR-449a inhibitor in the SCI *in vitro* model.

TNIP2, a binding partner of A20, is a negative mediator of NF-κB signaling [32]. Previous reports have revealed that the NF-κB signaling pathway is activated during SCI [55]. Therefore, in the present study we investigated whether the NF-κB pathway is involved in the regulatory mechanism of miR-449a in an SCI model. We observed that NF-κB signaling was activated in LPS-stimulated PC-12 cells, whereas miR-449a silencing significantly blocked the activation of the NF-κB pathway. However, TNIP2 knockdown reversed these effects.

Taken together, the present study indicated that lncRNA-SNHG7 is involved in SCI by regulating the miR-449a/TNIP2/NF-κB signaling pathway. However, this study did not investigate the role and mechanism of lncRNA-SNHG7 in primary neuronal inflammatory injury. This was a limitation of current study, and we will further perform this investigation in the future.

## Conclusion

The results of this study suggest that lncRNA-SNHG7 inhibits apoptosis and inflammatory responses in an SCI *in vitro* model by regulating the miR-449a/TNIP2/NF-κB signaling pathway. Therefore, SNHG7/miR-449a may be a latent, effective therapeutic candidate for SCI.

## Data Availability

The datasets used and/or analyzed during the current study are available from the corresponding author on reasonable request.

## Research highlights

lncRNA-SNHG7 was downregulated, and miR-499a was significantly upregulated in the in vivo and in vitro models of SCI;

TNIP2 directly interacts with miR-449a;

The miR-449a inhibitor suppressed LPS-stimulated PC-12 cell damage by targeting TNIP2.
